# Sodium Danshensu Inhibits Oral Cancer Cell Migration and Invasion by Modulating p38 Signaling Pathway

**DOI:** 10.3389/fendo.2020.568436

**Published:** 2020-09-30

**Authors:** V. Bharath Kumar, Shu-Hui Lin, B. Mahalakshmi, Yu-Sheng Lo, Chia-Chieh Lin, Yi-Ching Chuang, Ming-Ju Hsieh, Mu-Kuan Chen

**Affiliations:** ^1^Department of Medical Laboratory Science and Biotechnology, Asia University, Taichung, Taiwan; ^2^Department of Surgical Pathology, Changhua Christian Hospital, Changhua, Taiwan; ^3^Department of Medical Laboratory Science and Biotechnology, Central Taiwan University of Science and Technology, Taichung, Taiwan; ^4^Institute of Research and Development, Duy Tan University, Da Nang, Vietnam; ^5^Oral Cancer Research Center, Changhua Christian Hospital, Changhua, Taiwan; ^6^Institute of Medicine, Chung Shan Medical University, Taichung, Taiwan; ^7^Department of Holistic Wellness, Mingdao University, Changhua, Taiwan; ^8^Graduate Institute of Biomedical Sciences, China Medical University, Taichung, Taiwan; ^9^Department of Otorhinolaryngology, Head and Neck Surgery, Changhua Christian Hospital, Changhua, Taiwan

**Keywords:** sodium danshensu (Pubchem CID: 23711819), oral squamous cell carcinoma (OSCC), P38 signaling pathway, migration, invasion

## Abstract

**Background:** Oral squamous cell carcinoma (OSCC) that comprises about 90% of all oral cancer cases is associated with poor prognosis due to its highly metastatic nature. The majority of OSCC treatment options are related detrimental side-effects.

**Hypothesis/Purpose:** The present study aimed at deciphering the effects of a bioactive phytochemical, sodium danshensu, on human oral cancer cell metastasis.

**Methods and Results:** The treatment of FaDu and Ca9-22 cells with different doses of sodium danshensu (25, 50, and 100 μM) caused a significant reduction in cellular motility, migration, and invasion, as compared to the untreated cells. This effect was associated with a reduced expression of MMP-2, vimentin and N-cadherin, together with an enhanced expression of E-cadherin and ZO-1. Further investigation on the molecular mechanism revealed that treatment with sodium danshensu caused significant reduction in p38 phosphorylation; however, phosphorylation of ERK1/2 significantly decreased only in FaDu cells, whereas p-JNK1/2 did not show any alteration. A combination of p38 and JNK1/2 inhibitors with sodium danshensu also reduced the migration in the FaDu and Ca9-22 cell lines.

**Conclusion:** Collectively, the present study findings reveal that sodium danshensu execute anti-metastatic effect by suppressing p38 phosphorylation in human oral cancer. The study identifies sodium danshensu as a potential natural anticancer agent that can be used therapeutically to manage highly metastatic OSCC.

## Introduction

Oral cancer is the 6th most common cancer worldwide with a 5-year survival rate of 50%, and about 90% of all oral cancer cases are considered as oral squamous cell carcinoma (OSCC) (1). In particular, squamous cell carcinoma that develops in the oral mucosal epithelium is a fatal condition because of highly invasive nature of the tumor and higher risk of cervical lymph node as well as distant organ metastases (2). Regarding therapeutic strategies, early-stage OSCC is primarily treated with surgery; whereas, for advanced-stage cancer patients with multiple lymph nodes or distant organ metastases, surgery is mostly accompanied with radiotherapy or chemotherapy (3). Despite considerable advancement, the majority of OSCC treatment options is associated with detrimental side-effects and can cause damage to surrounding normal tissues. Therefore, it is of prime importance to identify natural bioactive compounds with minimal or no side-effects that can effectively improve the prognosis of OSCC.

Danshensu or salvianic acid A is a bioactive, water soluble, phenolic compound found in *Salvia miltiorrhiza (Danshen)*, which is a traditional Chinese herb used extensively to treat patients with cardiovascular and cerebrovascular diseases and hyperlipidemia (4). It has been found that danshensu exerts cardioprotective effects against isoproterenol-induced myocardial ischemia primarily by inhibiting L-type calcium channel and reducing the contraction of cardiac muscle (5). Danshensu has also been found to exert preventive effects against γ radiation-induced human fetal liver cell death by reducing reactive oxygen species (ROS) production, upregulating antioxidant defense mechanism, inhibiting intrinsic apoptotic pathway, and increasing intracellular survival pathways (6). Similarly, danshensu has been found to exert anti-inflammatory effects against ultraviolet B radiation-induced corneal injury. The study findings have shown that danshensu prevents leukocyte influx by inhibiting IL-1β, IL-6, tumor necrosis factor-α, and monocyte chemoattractant protein-1, leading to improvement in corneal structural integrity and protection against radiation-induced corneal damage (7). Regarding neuroprotective effects (8), it has been found that danshensu can restore the cerebral blood flow after focal cerebral ischemia by increasing neurogenesis, inducing new artery formation, and increasing the diameter of collateral arteries (9). The wound healing activity of danshensu has been identified in normal human fibroblasts, where danshensu has been found to increase cell proliferation and collagen synthesis (10).

There is a growing pool of evidence suggesting the anti-cancer effects of danshen extracts (11–13). For instance, danshen extract has been shown to inhibit breast cancer cell proliferation by decreasing AKT phosphorylation and increasing p27 expression (14). The bioactive, lipid soluble, phenanthrene compounds (tanshinones) present in danshen have shown to inhibit human prostate cancer cell proliferation by inducing cell cycle arrest and apoptosis (15). Moreover, these compounds have been shown to inhibit human lung cancer cell proliferation by reducing the expressions of VEGF, cyclin A, and cyclin B (16, 17). Despite having evidence regarding anti-proliferative effects of danshen extracts on different cancer types, studies investigating the effects of specific bioactive compounds of danshen on cancer cell migration and invasion are scanty.

Since tumor invasion and distant metastasis are the main causative factors behind cancer-related poor prognosis, the present study was designed to investigate the effects of sodium danshensu on human tongue squamous carcinoma cell migration and invasion. The mechanism by which sodium danshensu exerts its anti-cancer effects was also investigated.

## Materials and Methods

### Cell Culture

The human tongue squamous carcinoma cell line, FaDu, was obtained from ATCC (Manas, VA). Another oral cancer cell line, Ca9-22, was obtained from and authenticated by the Japanese Collection of Research Bioresources Cell Bank (JCRB, Shinjuku, Japan). Dulbecco's Modified Eagle Medium (DMEM; Life Technologies, Grand Island, NY) containing nutritional supplements (as described previously) was used to culture the cells (18). The cells were kept inside an incubator, under humidified condition (temp: 37°C; CO_2_: 5%).

### Materials

**Figure 1:**
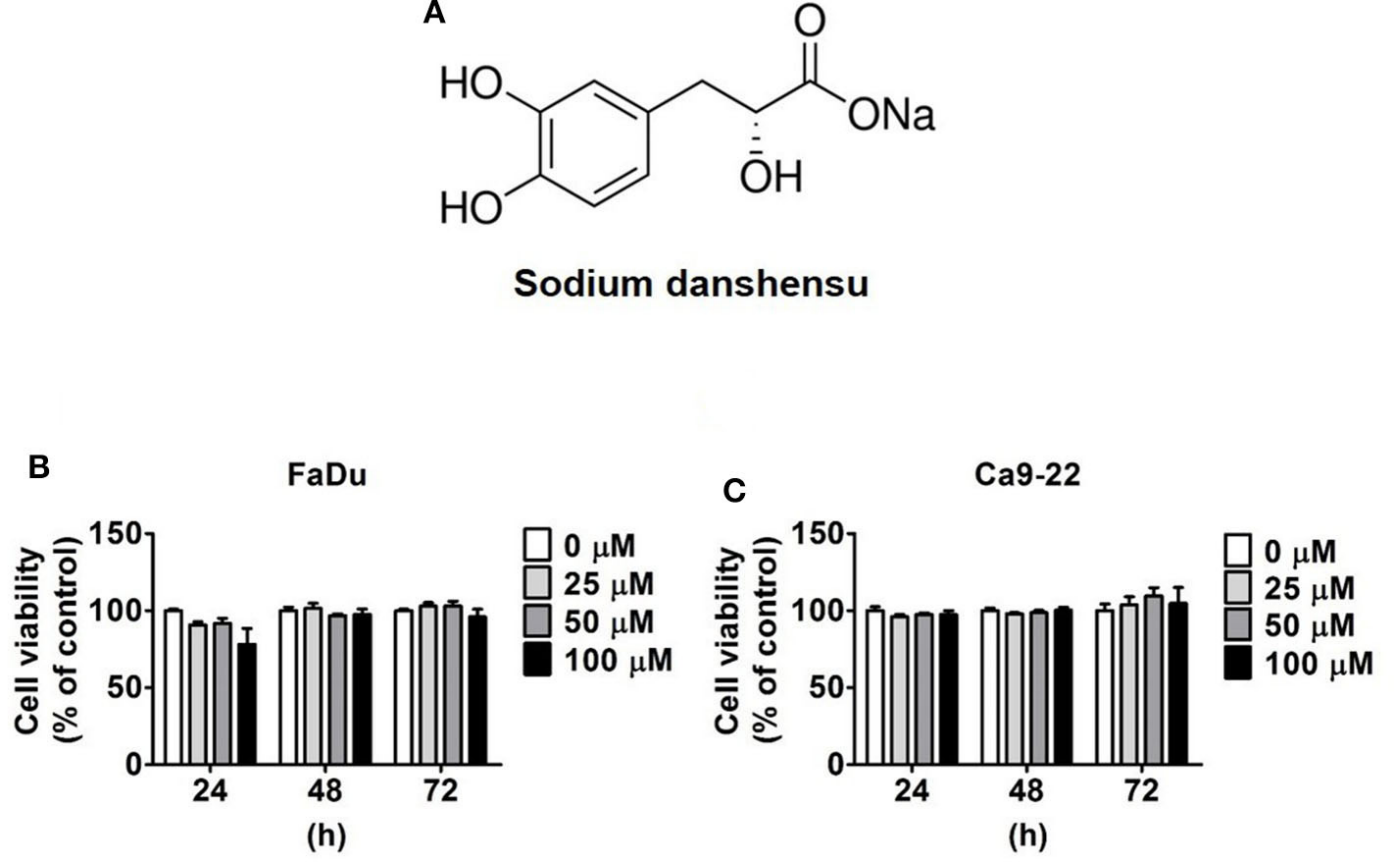
Cytotoxicity of sodium danshensu on human oral cancer cells. **(A)** The chemical structure of sodium danshensu (SD). **(B)** FaDu and **(C)** Ca9-22 cell lines were treated with various concentrations (0, 25, 50, and 100 μM) of sodium danshensu for 24, 48, and 72 h, and the cell viability was determined by MTT assay. The values are represented as mean ± SD of at least three independent experiments.

Sodium danshensu (≥98% purity) was purchased from ChemFaces (CheCheng Rd, Wuhan, PRC; Figure 1A). The stock solution (100 mM) of sodium danshensu was made in dimethyl sulfoxide (DMSO) and stored at −20°C. For all the treatments, DMSO final concentration was maintained at <0.2%. To obtain desired drug concentrations, the culture medium was supplemented with suitable amounts of sodium danshensu solution. Antibodies and specific MAPK pathway inhibitors (U0126, SB203580 and SP600125,) were purchased from Cell Signaling Technology, Inc. (Danvers, MA, USA) and Santa Cruz Biotechnology (Santa Cruz, CA), respectively.

### MTT Assay

The viability of FaDu and Ca9-22 cells was determined using MTT assay. For the experiment, the cells were seeded onto 24-well plates and treated with different concentrations of sodium danshensu (0–100 μM) for 24 h. After the treatments, the cell viability was assessed using MTT (3-(4,5-dimethylthiazol-2-yl)-2,5-diphenyltetrazolium bromide) assay, as defined earlier (19).

### Wound Healing Assay

For self-insertion, appropriate amounts of FaDu and Ca9-22 cells were seeded onto culture-insert wells (Ibidi, Martinsried, Germany) and incubated overnight. Next, the cells were treated with 0, 25, 50, and 100 μM of sodium danshensu for 0, 3, 6, 8, and 24 h after creating the wound. The cells were then photographed and the mean crawling distance was measured.

### Cell Migration and Invasion Assay

Trans-well assay was carried out to examine the migratory and invasive properties of FaDu and CA9-22 cells (Greiner Bio-One, North Carolina, USA). The cells were seeded onto transwell inserts (the upper well containing serum-free medium), incubated for 24 h, and allowed to migrate, as defined earlier (18, 20). Next, the cells were examined and photographed under a light microscope for the analysis of cellular migration and invasion.

### Western Blot Assay

The protein samples were extracted from the cells using lysis buffer, followed by separation using 10% polyacrylamide gel and transfer onto polyvinylidene fluoride (PVDF) membranes (Millipore Corporation, Milford, MA). The membranes were then blocked using 5% non-fat milk prepared in TBST for 1 h, followed by 24 h of incubation with specified primary antibodies at 4°C. Next, 1 h of secondary antibody (peroxidase-conjugated) incubation was done in room temperature. Finally, the protein bands were assessed using ECL detection system.

### Statistical Analysis

The data were collected from three independent experiments and represented as mean ± SD. The Student's *t*-test was used to analyze the experimental findings (SigmaPlot 10.0, San Jose, California). A *p* < 0.05 was considered as statistically significant.

## Results

### Cytotoxic Effect of Sodium Danshensu on Human Oral Cancer Cells

The cytotoxic effects of sodium danshensu on FaDu and Ca9-22 cells was examined using MTT assay. The cells were treated with different concentrations of sodium danshensu (25, 50, and 100 μM) for 24, 48, and 72 h, and untreated cells were used as control. As observed in Figures 1B,C, none of the doses of sodium danshensu caused any alteration in cell viability until 72 h of treatment. This finding indicates that sodium danshensu does not have any cytotoxic effect on human oral cancer cells.

### Effect of Sodium Danshensu on Motility of Human Oral Cancer Cells

**Figure 2:**
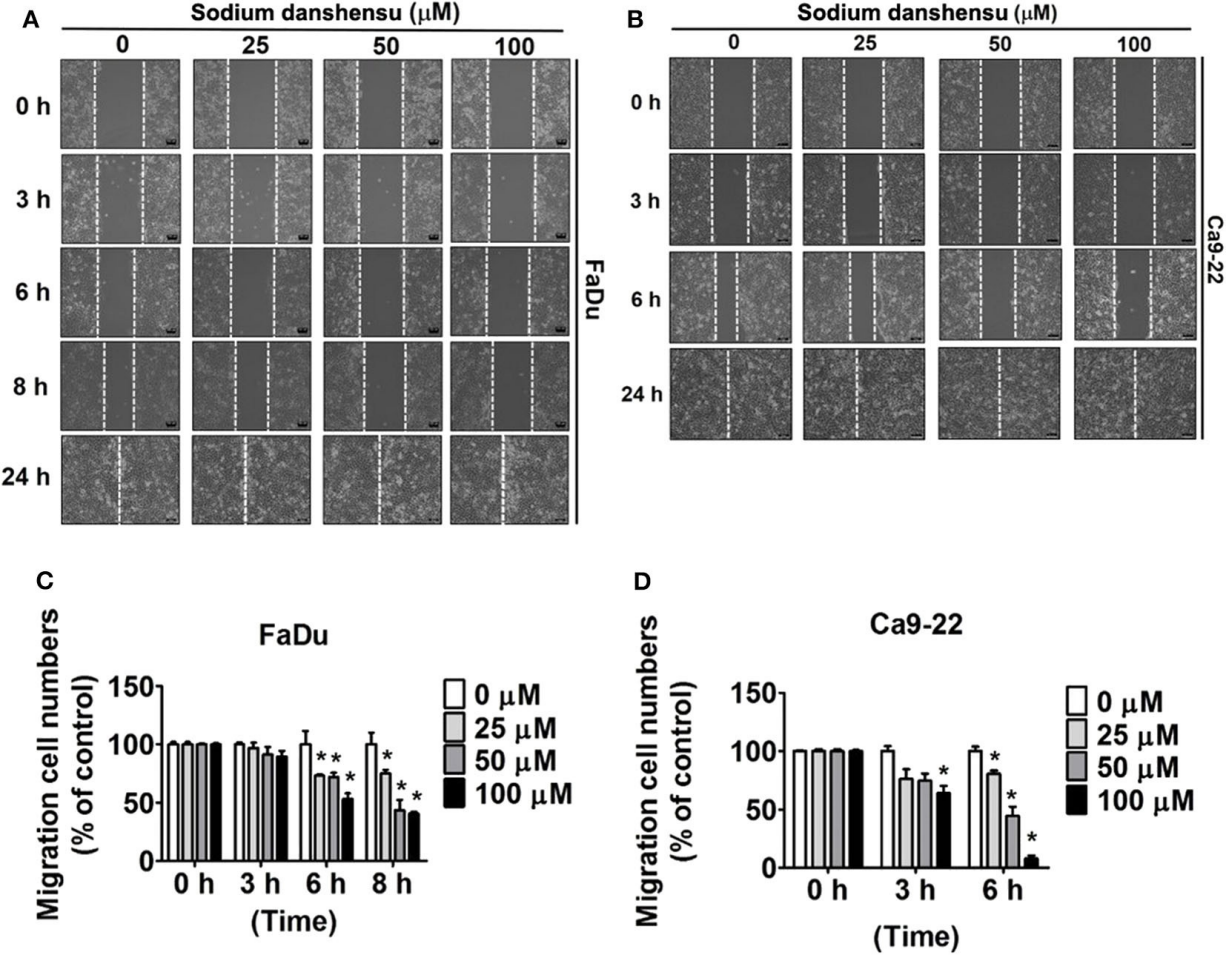
Sodium danshensu inhibits cell motility in human oral cancer cells. The effect of sodium danshensu treatment on cell motility was analyzed in **(A,B)** FaDu and **(C,D)** CA9-22cells using wound healing assay. The values are represented as mean ± SD of at least three independent experiments. *P < 0.05, compared to the control (no treatment) group.

To investigate the effect of sodium danshen on the motility of human oral cancer cells, wound healing assay was performed on FaDu and Ca9-22 cells. As observed in Figures 2A,B the motility of FaDu cells treated with sodium danshensu for 6 or 8 h caused significant reduction com reduced as compared to that of untreated cells. Similar trend was observed in sodium danshensu treated Ca9-22 cells for 6 h treatment (Figures 2C,D). Ca9-22 cells started to migrate into the scratched site at 8 h of incubation than FaDu cells. Based on the result of the wound healing assay, it was suggested that sodium danshensu can significantly reduce the motility of human oral cancer cells.

### Effect of Sodium Danshensu on Migration and Invasion of Human Oral Cancer Cells

**Figure 3:**
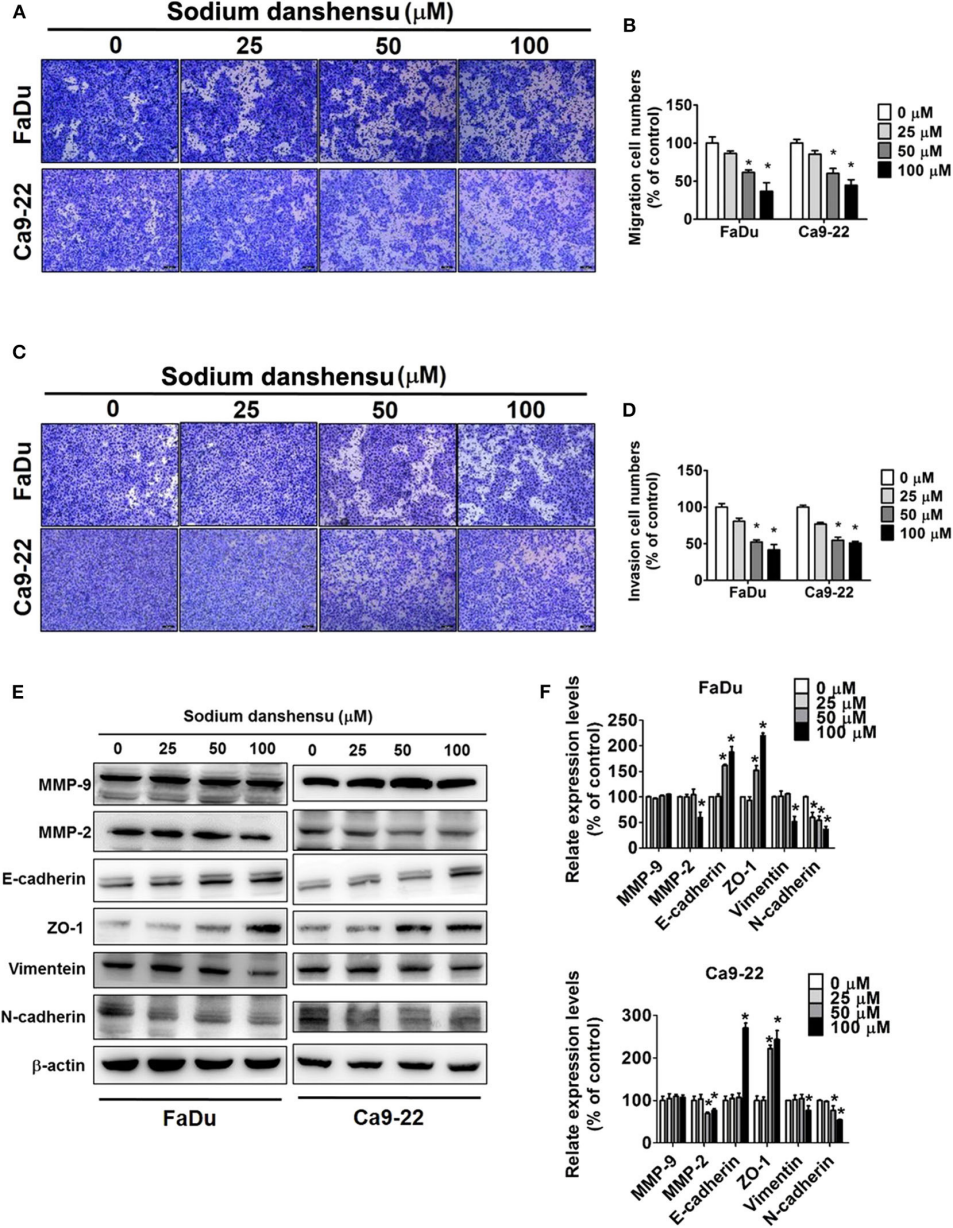
Sodium danshensu inhibits cell migration and invasion in human oral cancer cells. The effect of sodium danshensu treatment on cell migration **(A)** and invasion **(C)** was measured using trans-well assay in FaDu and Ca9-22 cells. The percentages of cells in migration and invasion assays are shown in **(B,D)**, respectively. Sodium danshensu treatment-induced changes in MMP-9, MMP-2, E-cadherin, ZO-1, vimentein and N-cadherin **(E,F)** were measured in FaDu and Ca9-22 cell lines using Western blot analysis. The values are represented as mean ± SD of at least three independent experiments. *P < 0.05, compared to the control group.

Next, the investigation of the effect of sodium danshensu on migration and invasion of FaDu and Ca9-22 cells was done using transwell assay. The cells were treated with 25, 50, and 100 μM of sodium danshensu for 24 h for the assay. As observed in Figures 3A,B, sodium danshensu at higher concentrations (50 and 100 μM) caused significant reduction in migration of both FaDu and Ca9-22 cells, as compared to untreated control cells. Similarly, compared to the control, the invasion of FaDu and Ca9-22 cells was decreased significantly after the treatment with 50 and 100 μM of sodium danshensu (Figures 3C,D).

To explore the mechanism underlying the anti-cancer effect of sodium danshensu, western blotting analysis was applied to evaluate the regulating effect of sodium danshensu on the EMT markers. We observed that the expression of MMP-2, vimentin and N-cadherin in sodium danshensu-treated cells was inhibited at a greater extent than control cells (Figures 3E,F). Under the same experimental condition, the expression of E-cadherin and ZO-1 was increased in sodium danshensu treated FaDu and Ca9-22 cells. No significant changes were found in MMP-9 expression. These findings clearly demonstrate the anti-migratory and anti-invasive effects of sodium danshensu on human oral cancer cells.

### Effect of Sodium Danshensu on Mitogen-Activated Protein Kinases (MAPKs) in Human Oral Cancer

**Figure 4:**
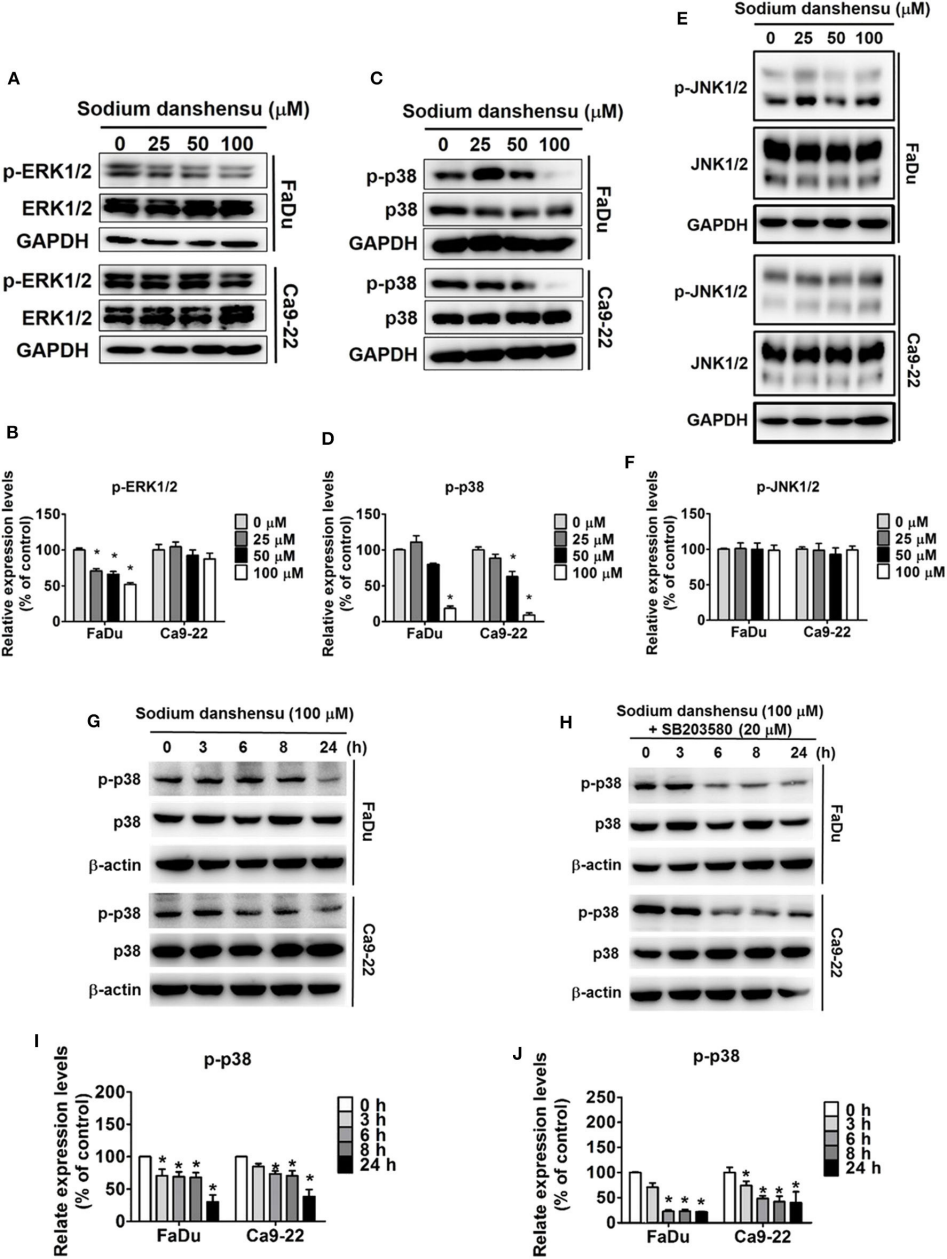
Sodium danshensu inhibits p38 pathway in human oral cancer cells. Sodium danshensu treatment-induced changes in phosphorylation status as well as total protein expressions of ERK1/2 **(A,B)**, p38 **(C,D)**, and JNK **(E,F)** were measured in FaDu and Ca9-22 cell lines using Western blot analysis. Sodium danshensu treatment-induced time course changes in phosphorylation status of p38 **(G,H)**, and the impact of SB203580 and sodium danshensu co-treatment on the p38 phosphorylation during a time course **(I,J)** were measured in FaDu and Ca9-22 cell lines using Western blot analysis. The values are represented as mean ± SD of at least three independent experiments. *P < 0.05, compared to the control group.

Next, the mode of action of sodium danshensu in regulating cell migration and invasion was investigated. Given the significant involvement of MAPKs in controlling cell migration (16), we thought of studying the role of sodium danshensu in regulating the expression and activity of MAPKs, including ERK1/2, p38, and JNK. As observed in Figures 4A,B, phosphorylation of ERK reduced dose-dependently after 24 h of sodium danshensu treatment. However, no such effect was observed in Ca9-22 cells. The maximum change was observed in case of p38, where sodium danshensu treated caused significant reduction in p38 phosphorylation in both the cell lines (Figures 4C,D). In case of JNK, no change was observed following sodium danshensu treatment (Figures 4E,F).

We then validated the effect of sodium danshensu on p38 expression in a time dependent manner. Human oral cancer cells -FaDu and Ca9-22 cells were treated sodium danshensu (100 μM) and then analyzed for p38 phosphorylation expression by western blotting. As shown in Figures 4G,H, sodium danshensu time dependently decreased p-p38 expression in both cell lines. Based on this result we further treated FaDu and Ca9-22 cells with SB203580 (p38 inhibitor- SB203580) and 100 μM of sodium danshensu for 3, 6, 8, and 24 h and then analyzed for p-p38 expression (Figures 4I,J). Combinatorial treatment with p38 inhibitor and sodium danshensu greatly reduced p-p38 expression in FaDu and Ca9-22 cells. These results indicate that sodium danshensu cooperatively downregulates p-p38 and inhibits the MAPK signaling pathway along with p38 inhibitors in oral cancer cells.

### Effect of SB203580, U0126, or SP600125 and Sodium Danshensu Co-treatment on Cell Motility, Migration and Invasion in Human Oral Cancer Cells

**Figure 5:**
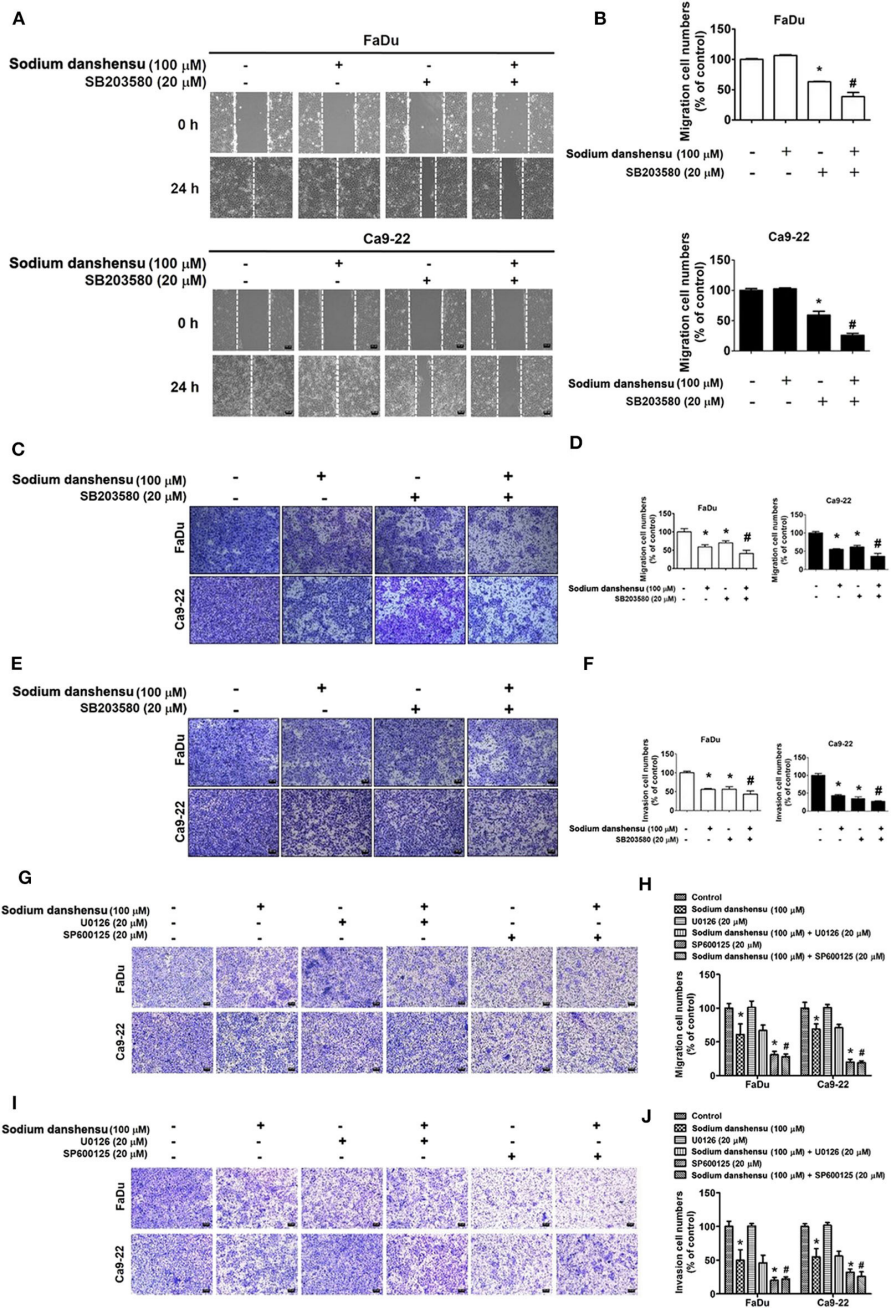
Effect of SB203580 and sodium danshensu co-treatment on cell motility, migration and invasion in FaDu and Ca9-22 cell lines. The cell motility was measured using wound closer assay **(A,B)** after the co-treatment with SB203580 and sodium danshensuin FaDu and Ca9-22 cell lines. The cell migration **(C,D)** and invasion **(E,F)** were measured using trans-well assay after the co-treatment with SB203580 and sodium danshensuin FaDu and Ca9-22 cell lines. The cell migration **(G,H)** and invasion **(I,J)** were measured using trans-well assay after the co-treatment with U0126 or SP600125 and sodium danshensu in FaDu and Ca9-22 cell lines. The values are represented as mean ± SD of at least three independent experiments. *P < 0.05, compared to the control group; #*p* < 0.05, compared to the sodium danshensu-treated group.

To further establish the involvement of p38 signaling in sodium danshensu-induced anti-migratory effects, we co-treated FaDu and Ca9-22 cells with sodium danshensu and SB203580, which is a selective p38 inhibitor. As shown in Figures 5A,B, the co-treatment further reduced the cell motility, as compared to sodium danshensu treatment alone. Similarly, the co-treatment caused further reduction in cellular migration and invasion, as compared to both untreated and sodium danshensu-treated cells (Figures 5C–F).

We then verified the involvement of ERK or JNK expression in sodium danshensu induced antimigratory effect. FaDu and Ca9-22 cells were co-treated with sodium danshensu and U0126 (ERK inhibitor) or SP600125 (JNK inhibitor) and analyzed for migration and invasion ability through transwell method. Results showed that, combinatory treatment no significantly reduced cell migration (Figures 5G,H) and invasion (Figures 5I,J) of sodium danshensu-treatment FaDu and Ca9-22 cells in transwell assay.

## Discussion

The present study was designed to investigate the effect of sodium danshensu, which is a water soluble, phenolic compound found in a traditional Chinese medicinal plant Salvia miltiorrhiza (Danshen), on cancer cell migration and invasion in human tongue squamous cell carcinoma. As mentioned earlier, the anti-cancer properties of Danshen extracts observed in previous studies are mostly associated with inhibition of cellular proliferation and induction of apoptosis 14, 15. To the best of our knowledge, this is the first study to demonstrate the anti-migratory and anti-invasive properties of water soluble, bioactive phytochemical, sodium danshensu, in human tongue squamous cell carcinoma.

To conduct the experiments, we treated FaDu and Ca9-22 cells with different concentrations of sodium danshensu (25, 50, and 100 μM) for indicated time points. The MTT assay conducted for investigating the cytotoxic effects, and our results showed that sodium danshensu does not affect the viability of human oral cancer cells for 72 h (Figures 1B,C), which is not in line with previous observations showing anti-proliferative effects of Danshen extracts on different cancer types (21). This discrepancy in observations may be because of the differences in molecular characteristics between compounds used in previous studies. For example, the anti-proliferative effects have mostly been observed for lipid soluble, phenanthrenes present in Danshen extracts; in contrast, we used a water soluble, phenolic compound in the present study. Interestingly, one recent study conducted on Lewis lung carcinoma xenograft mouse model has shown that treatment with danshensu significantly increases the growth inhibitory effects of radiation therapy most probably by improving tumor vasculature and eliminating tumor hypoxia (22).

Regarding cancer cell invasion and migration, the findings of wound closer as well as transwell assays clearly demonstrate that sodium danshensu significantly decreases the motility, migration, and invasion of human oral cancer cells, resulting in reduced cellular ability to migrate and invade. These findings are in line with previous studies showing that tanshinone IIA compound of Danshen can inhibit the migration and invasion of different cancer types, including colon, liver, and breast cancers (23). EMT is characterized by the loss of cell adhesion molecules, like E-cadherin and increase of mesenchymal markers such as vimentin (24). We further quantified the effects of sodium danshensu on EMT by examining the expression of several key epithelial and mesenchymal markers. Sodium danshensu treatment significantly increased protein levels of the epithelial markers- E-cadherin and ZO-1 and decreased protein levels of the mesenchymal markers N-cadherin and vimentin compared with the control group. Furthermore, sodium danshensu treatment decreased MMP-2 expression without altering MMP-9 expression in both these cancer cells. Similar results were observed in pinostilbene hydrate (PSH) treated oral cancer- SCC9 and SAS cell lines (18). These results are contradictory from the previous data, Tan-IIA treatment decreased both MMP-2 and−9 AGS cells (25). This might be due to differences in molecular characteristics between compounds.

The MAPK inhibitory effects of active Danshen components have been observed previously. For instance, salvianolic acid A has been found to reduce kidney damage in nephrectomized mice by inhibiting NFκB activation and p38 phosphorylation, which in turn lead to attenuation of cellular inflammatory response (26). Similarly, salvianolic acid B has been found to exert anti-fibrotic effects in human hepatic stellate cells by attenuating p38 and ERK signaling pathways and inhibiting the crosstalk between Smad and ERK (27). In cardiac fibrosis, danshensu has been found to inhibit β-adrenergic receptor-mediated fibroblast proliferation and collagen-I synthesis primarily by suppressing p38 signaling pathway (28). Recently, one study has shown than salvianolic acid A can prevent OSCC metastasis by inhibiting Raf/MEK/ERK signaling pathway, which subsequently causes reduction of MMP-2 expression (29). To investigate the molecular mechanism involved in sodium danshensu-mediated anti-metastatic effects, we thought of examining the level and activity of conventional MAPKs, including ERK1/2, JNK1/2, and p38. The Western blot analysis data revealed that treatment with sodium danshensu causes significant reduction in p38 phosphorylation in both FaDu and Ca9-22 cells in a dose and time dependent manner (Figures 4C,G). The phosphorylation of ERK1/2 also showed the similar trend, but only in FaDu cells. However, no change in JNK1/2 phosphorylation was found in either of the cell lines (Figures 4E,F). To further confirm that sodium danshensu inhibits cell migration by p38 MAPK pathway, we used p38 inhibitor -SB203580 alone or in combination with sodium danshensu and analyzed for p-p38 expression in oral cancer cells. Sodium danshensu combination with SB203580, markedly reduces the p-p38 expression (Figures 4G–J). Because sodium danshensu doesn't inhibit ERK1/2 and JNK1/2 expression, we further dint evaluate the time dependent effect of drugs on these proteins. These observations highlight the involvement of p38 signaling pathway in sodium danshensu-mediated anti-metastatic effects in human OSCC.

The final affirmation on the involvement of p38 pathway came from the observations demonstrating that the cotreatment with sodium danshensu and a selective p38 inhibitor, SB203580, causes further reduction in motility and metastatic behaviors of FaDu and Ca9-22 cells, as compared to sodium danshensu treatment alone (Figures 5A–F).

In conclusion, the present study demonstrates the effect of sodium danshensu, which is a bioactive, water soluble, phenolic compound found in *Salvia miltiorrhiza (Danshen)*, on cancer cell migration and invasion in human OSCC. The study findings reveal that sodium danshensu significantly reduces the motility and metastatic ability of oral cancer cells by reducing the phosphorylation of p38 MAPK. However, sodium danshensu-mediated alteration in ERK1/2 phosphorylation was observed only in FaDu cells. Collectively, the present study identifies sodium danshensu as a potential natural anticancer agent that can be used therapeutically to manage highly metastatic OSCC.

## Data Availability Statement

The original contributions presented in the study are included in the article/supplementary material, further inquiries can be directed to the corresponding author/s.

## Author Contributions

M-JH and S-HL: conceptualization. Y-SL, C-CL, and Y-CC: methodology and software. VK and BM: writing–original draft preparation. M-JH and M-KC: writing–review and editing. All authors read and approved the final manuscript.

## Conflict of Interest

The authors declare that the research was conducted in the absence of any commercial or financial relationships that could be construed as a potential conflict of interest.

## Abbreviations

ERKs: Extracellular signal–regulated kinases
JNKs: c-Jun N-terminal kinases
MAPK: Mitogen-activated protein kinases.

## References

[1] Markopoulos AK. Current aspects on oral squamous cell carcinoma. Open Dent J. (2012) 6:126–30. 10.2174/1874210601206010126 22930665 PMC3428647

[2] Thomson PJ. Perspectives on oral squamous cell carcinoma prevention-proliferation, position, progression and prediction. J Oral Pathol Med. (2018) 47:803–7. 10.1111/jop.12733 29752860

[3] Omura K. Current status of oral cancer treatment strategies: surgical treatments for oral squamous cell carcinoma. Int J Clin Oncol. (2014) 19:423–30. 10.1007/s10147-014-0689-z 24682763

[4] Wang L Ma R Liu C Liu H Zhu R Guo S. *Salvia miltiorrhiza*: a potential red light to the development of cardiovascular diseases. Curr Pharm Des. (2017) 23:1077–97. 10.2174/1381612822666161010105242 27748194 PMC5421141

[5] Song Q Chu X Zhang X Bao Y Zhang Y Guo H. Mechanisms underlying the cardioprotective effect of Salvianic acid A against isoproterenol-induced myocardial ischemia injury in rats: possible involvement of L-type calcium channels and myocardial contractility. J Ethnopharmacol. (2016) 189:157–64. 10.1016/j.jep.2016.05.038 27211016

[6] Guo J Zhang Y Zeng L Liu J Liang J Guo G. Salvianic acid A protects L-02 cells against gamma-irradiation-induced apoptosis via the scavenging of reactive oxygen species. Environ Toxicol Pharmacol. (2013) 35:117–30. 10.1016/j.etap.2012.11.010 23274418

[7] Teng MC Wu PC Lin SP Wu CY Wang PH Chen CT. Danshensu decreases UVB-induced corneal inflammation in an experimental mouse model via oral administration. Curr Eye Res. (2018) 43:27–34. 10.1080/02713683.2017.1379543 29111819

[8] Guo C Yin Y Duan J Zhu Y Yan J Wei G. Neuroprotective effect and underlying mechanism of sodium danshensu [3-(3,4-dihydroxyphenyl) lactic acid from radix and rhizoma *Salviae miltiorrhizae* = danshen] against cerebral ischemia and reperfusion injury in rats. Phytomedicine. (2015) 22:283–9. 10.1016/j.phymed.2014.12.001 25765834

[9] Wei ZZ Chen D Liu LP Gu X Zhong W Zhang YB. Enhanced neurogenesis and collaterogenesis by sodium danshensu treatment after focal cerebral ischemia in mice. Cell Transplant. (2018) 27:622–36. 10.1177/0963689718771889 29984620 PMC7020234

[10] Chen YS Lee SM Lin YJ Chiang SH Lin CC. Effects of danshensu and salvianolic acid B from *Salvia miltiorrhiza* bunge (Lamiaceae) on cell proliferation and collagen and melanin production. Molecules. (2014) 19:2029–41. 10.3390/molecules19022029 24531218 PMC6271020

[11] Lin YY Lee IY Huang WS Lin YS Kuan FC Shu LH. Danshen improves survival of patients with colon cancer and dihydroisotanshinone I inhibit the proliferation of colon cancer cells via apoptosis and skp2 signaling pathway. J Ethnopharmacol. (2017) 209:305–16. 10.1016/j.jep.2017.08.011 28807849

[12] Lin YS Shen YC Wu CY Tsai YY Yang YH Lin YY. Danshen improves survival of patients with breast cancer and dihydroisotanshinone i induces ferroptosis and apoptosis of breast cancer cells. Front Pharmacol. (2019) 10:1226. 10.3389/fphar.2019.01226 31736748 PMC6836808

[13] Wang T Fu X Wang Z. Danshen formulae for cancer: a systematic review and meta-analysis of high-quality randomized controlled trials. Evid Based Complement Alternat Med. (2019) 2019:2310639. 10.1155/2019/2301680 31061667 PMC6466905

[14] Yang W Ju JH Jeon MJ Han X Shin I. Danshen (*Salvia miltiorrhiza*) extract inhibits proliferation of breast cancer cells via modulation of Akt activity and p27 level. Phytother Res. (2010) 24:198–204. 10.1002/ptr.2945 19610045

[15] Gong Y Li Y Lu Y Li L Abdolmaleky H Blackburn GL. Bioactive tanshinones in *Salvia miltiorrhiza* inhibit the growth of prostate cancer cells in vitro and in mice. Int J Cancer. (2011) 129:1042–52. 10.1002/ijc.25678 20848589 PMC3032031

[16] Huang C Jacobson K Schaller MD. MAP kinases and cell migration. J Cell Sci. (2004) 117:4619–28. 10.1242/jcs.01481 15371522

[17] Tung YT Chen HL Lee CY Chou YC Lee PY Tsai HC. Active Component of Danshen (*Salvia miltiorrhiza* Bunge), tanshinone I, Attenuates lung tumorigenesis via inhibitions of VEGF, cyclin A, and cyclin B expressions. Evid Based Complement Alternat Med. (2013) 2013:319247. 10.1155/2013/319247 23662128 PMC3638627

[18] Hsieh MJ Chin MC Lin CC His YT Lo YS Chuang YC. Pinostilbene hydrate suppresses human oral cancer cell metastasis by downregulation of matrix metalloproteinase-2 through the mitogen-activated protein kinase signaling pathway. Cell Physiol Biochem. (2018) 50:911–23. 10.1159/000494476 30355929

[19] Wei CW Lin CC Yu YL Lin CY Lin PC Wu MT. n-Butylidenephthalide induced apoptosis in the A549 human lung adenocarcinoma cell line by coupled down-regulation of AP-2alpha and telomerase activity. Acta Pharmacol Sin. (2009) 30:1297–306. 10.1038/aps.2009.124 19701232 PMC4007174

[20] Ho HY Ho YC Hsieh MJ Yang SF Chuang CY Lin CW. Hispolon suppresses migration and invasion of human nasopharyngeal carcinoma cells by inhibiting the urokinase-plasminogen activator through modulation of the Akt signaling pathway. Environ Toxicol. (2017) 32:645–55. 10.1002/tox.22266 27037602

[21] Wang WH Hsuan KY Chu LY Lee CY Tyan YC Chen ZS Anticancer effects of *Salvia miltiorrhiza* alcohol extract on oral squamous carcinoma cells. Evid Based Complement Alternat Med. (2017) 2017:5364010 10.1155/2017/5364010 28246540 PMC5303586

[22] Cao HY Ding RL Li M Yang MN Yang LL Wu JB. Danshensu, a major water-soluble component of *Salvia miltiorrhiza*, enhances the radioresponse for lewis lung carcinoma xenografts in mice. Oncol Lett. (2017) 13:605–12. 10.3892/ol.2016.5508 28356936 PMC5351344

[23] Zhang Y Jiang P Ye M Kim SH Jiang C Lu J. Tanshinones: sources, pharmacokinetics and anti-cancer activities. Int J Mol Sci. (2012) 13:13621–66. 10.3390/ijms131013621 23202971 PMC3497345

[24] Robert G Gaggioli C Bailet O Chavey C Abbe P Aberdam E. SPARC represses E-cadherin and induces mesenchymal transition during melanoma development. Cancer Res. (2006) 66:7516–23. 10.1158/0008-5472.CAN-05-3189 16885349

[25] Su CC. Tanshinone IIA decreases the migratory ability of AGS cells by decreasing the protein expression of matrix metalloproteinases, nuclear factor kappaB-p65 and cyclooxygenase-2. Mol Med Rep. (2016) 13:1263–8. 10.3892/mmr.2015.4658 26648518

[26] Zhang HF Wang YL Gao C Gu YT Huang J Wang JH. Salvianolic acid A attenuates kidney injury and inflammation by inhibiting NF-kappaB and p38 MAPK signaling pathways in 5/6 nephrectomized rats. Acta Pharmacol Sin. (2018) 39:1855–64. 10.1038/s41401-018-0026-6 29795135 PMC6289371

[27] Lv Z Xu L. Salvianolic acid B inhibits ERK and p38 MAPK signaling in TGF-beta1-stimulated human hepatic stellate cell line (LX-2) via distinct pathways. Evid Based Complement Alternat Med. (2012) 2012:960128. 10.1155/2012/960128 21860657 PMC3155803

[28] Lu H Tian A Wu J Yang C Xing R Jia P. Danshensu inhibits beta-adrenergic receptors-mediated cardiac fibrosis by ROS/p38 MAPK axis. Biol Pharm Bull. (2014) 37:961–7. 10.1248/bpb.b13-00921 24882408

[29] Fang CY Wu CZ Chen PN Chang YC Chuang CY Lai CT. Antimetastatic potentials of salvianolic acid A on oral squamous cell carcinoma by targeting MMP-2 and the c-Raf/MEK/ERK pathway. Environ Toxicol. (2018) 33:545–54. 10.1002/tox.22542 29385302

